# Estimation of breed effects and non-additive genetic variation for ostrich slaughter and skin traits

**DOI:** 10.1007/s11250-024-04168-8

**Published:** 2024-09-30

**Authors:** Khetho Ratshilumela Nemutandani, Anel Engelbrecht, Schalk Willem Petrus Cloete, Kennedy Dzama, Obert Tada

**Affiliations:** 1 Department of Animal Sciences, Stellenbosch University, Private Bag X1, Matieland, 7602 South Africa; 2 Department of Agricultural Economics and Animal Production, University of Limpopo, Private Bag X1106, Sovenga, 0727 South Africa; 3 Directorate Animal Sciences, Western Cape Department of Agriculture, Private Bag X1, Elsenburg, 7607 South Africa

**Keywords:** Crossbreeding, Heterosis, Purebred, Dam line effect

## Abstract

The study quantified breed effects and putative non-additive genetic variation for quantitative and qualitative slaughter and skin traits involving three ostrich breeds: South African Black (SAB), Zimbabwean Blue (ZB), and Kenyan Red (KR) ostriches. Such data from contemporary slaughter groups with all three pure breeds represented were analyzed together (SAB: *n* = 457; ZB: *n* = 74; KR: *n* = 50). Two 2 × 2 diallel crossbreeding designs were used to assess crosses of ZB and KR birds with the SAB strain. Subsequently, the data from SAB, ZB and their reciprocal crosses, slaughtered together; as well as the data from SAB and KR ostriches, along with their reciprocal crosses, respectively, were analyzed separately. ZB and KR birds outperformed SAB birds for most size-related slaughter and skin traits. Linear contrasts were used to distinguish the effects of breed, heterosis, and the dam line. For the ZB x SAB design, additive breed effects were significant for slaughter weight (10.4%), crust skin size (3.9%) and nodule shape score (1.6%) (all *P* < 0.05). Significant heterosis were found for slaughter weight (4.3%), crust skin size (1.7%) and nodule size score (3.7%) (*P < 0.05*). Dam line effects were observed for skin weight (4.1%) and hair follicle score (4.1%). In the KR x SAB design, additive breed effects were like outcomes for the ZB x SAB design. Heterosis estimates were significant (*P < 0.05*) for slaughter weight (5.8%), crust skin size (2.9%), crown length (1.7%) and nodule size score (4.4%). Dam line effects were significant for slaughter weight (3.7%) and nodule size score (3.8%). In conclusion, ZB and KR birds outperformed their SAB contemporaries for size and size-related traits. Crossbreeding with these genotypes could improve some size-related traits over the mid-parent value of purebreds.

**Supplementary Information:**

The online version contains supplementary material available at 10.1007/s11250-024-04168-8.

## Introduction

Domestication of ostriches in South Africa started more than 100 years ago with the focus on feather production (Douglass 1881; Smit 1964; Wagner 1986), resulting in the development of the ostrich farming industry (Deeming and Bubier 1999). The domesticated South African Black ostrich (*Struthio camelus* var. *domesticus*) was developed over time through a process of selection and crossbreeding (Osterhoff 1979). This is still the breed mostly farmed with in South Africa. According to Van Zyl (1996), the emphasis shifted away from feathers towards the production of leather by 1975 and by the late 1980’s the ostrich industry was well established and based on the slaughter of ostriches to produce leather, meat and feathers in descending order of importance (Van Zyl 2001). To retain competitiveness, the improvement of product quality is vital.

It is well-documented with other farm animals that crossbreeding can increase the performance of hybrids over the mid-parent value of pure breeds in commercial operations through heterosis (Bourdon 1997; Baranwal et al. 2012). It is thus not unreasonable to surmise that a structured crossbreeding plan may also assist in improving current traits of economic importance in the ostrich industry. Kawka et al. (2007) reported significant genetic distances among South African Black (SAB), Zimbabwean Blue (ZB) and Kenyan Red (KR) ostriches. Davids et al. (2012) subsequently performed genetic diversity studies within and between different ostrich genotypes and suggested that crossbreeding of the different genotypes could result in heterosis. Engelbrecht et al. (2008) reported that ZB and KR ostriches have indeed been introduced to some SAB ostrich flocks to improve slaughter production, albeit somewhat haphazardly, because the live weight of ZB and KR ostriches were demonstrated to be higher than that of SAB birds (Cloete et al. 2008; Engelbrecht et al. 2008). Engelbrecht et al. (2008) also reported that the introduction of Zimbabwean Blue ostriches to a SAB flock led to direct heterosis for growth. Hereafter, significant additive breed and heterosis effects were also reported for some slaughter and meat traits of SAB and ZB birds, and their reciprocal crosses, as well as for SAB and KR birds, and their reciprocal crosses, respectively (Engelbrecht et al. 2018). At this stage it is not known if the additive breed and heterosis effects reported for slaughter and meat traits will also be observed for skin (or leather) traits. In fact, due to the poor feather quality of ZB and particularly KR birds compared to that of SAB ostriches (Brand and Cloete 2015), it is feared that crossbreeding the SAB with either of these two breeds might negatively impact on leather quality. On the other hand, the high correlation between live weight and skin traits reported by Cloete et al. (1998) and Engelbrecht et al. (2007, 2009), suggests that improving live weight could potentially benefit most leather traits as correlated responses. Considering the importance of ostrich leather as a product for the South African industry, the effect of crossbreeding on skin quality needs to be investigated considering the current breeding practices (i.e. flock breeding) in the industry.

Against this background, it was hypothesised that slaughter and skin yield, and skin quality traits of different ostrich genotypes (SAB, ZB and KR) and their crosses will: (1) Display additive breed effects, as well as the expected non-additive genetic variation; and (2) that it will be possible to improve such traits can be improved by breed replacement and/or crossbreeding. These hypothesizes were formulated to provide information to the ostrich industry to scientifically guide decision-making on breed performance and crossbreeding.

## Materials and methods

### Genetic resources and data description

Slaughter and skin data analysed were from ostriches that were slaughtered from 2009 to 2020. The base population for the study was the ostrich resource flock kept at the Western Cape Department of Agriculture’s Oudtshoorn Research Farm in South Africa. The flock was started during 1964 from a donation of 76 SAB breeder birds from 61 local producers (Engelbrecht 2013). Birds from other ostrich genotypes, i.e. ZB (*n* = 27; 2003) and the KR (*n* = 18; 2007) ostriches, were later introduced into the resource flock (see Engelbrecht et al. 2008) to study breed effects. Given the dominance of SAB ostriches in the commercial ostrich industry, and the improved reproduction of SAB females relative to ZB females (Cloete et al. 2008; Cloete and Brand 2014), the SAB was identified as a potential dam line in a crossbreeding operation. The ZB and KR ostriches were consequently introduced to the flock in a structured crossbreeding program, involving the three pure bloodlines and the reciprocal crosses between the ZB and the SAB ostriches, as well as between the KR and the SAB ostriches, respectively.

The management of the research flock is well documented (Engelbrecht 2013). In short, individual breeding birds are kept as pairs in small camps of approximately 0.25 hectare. Eggs are collected daily and the date of lay, as well as the camp of origin are indicated on the shell. Eggs are incubated artificially and each chick that hatch is identified with a neck tag that is linked to the egg and camp of origin to ensure that parentage is known. Chicks are reared up to slaughter age in groups of approximately 100 chicks per group. Selected birds are kept as for breeding purposes, while the rest are slaughtered (Engelbrecht 2013). The ostriches are reared under feedlot conditions for most of their life, with ostrich diets (formulated according to growth stage) and potable water provided *ad libitum*.

Three data sets were prepared from the available slaughter data, to represent the respective breed combinations and their reciprocal crosses. Breed effects were assessed in a complete randomised design (Snedecor and Cochran 1968). Crossbreeding effects were assessed in a 2 × 2 diallel crossbreeding design, allowing for the estimation of independent effects as described by Dickerson (1969). Limited birds were available for slaughter, ranging from 50 for the pure KR genotype to 519 for the SAB genotype analysed with the ZB (Table 1). Chick rearing facilities do not allow the rearing of all hatched chicks on the farm, while approximately 15–20% of birds were selected annually for breeding. Unfortunately, due to the comparatively small number of ZB and KR birds that were introduced to the flock, the number of slaughter records for ZB, KR and crossbreeding progeny were relatively small (Table 1).

**Table 1:** Number of records (N) for the pure breed (South African Black (SAB), Zimbabwean Blue (ZB) and Kenyan Red (KR) ostriches) analysis, as well as the respective crossbreeding analyses (SAB, ZB and their reciprocal crosses, and SAB, KR and their reciprocal crosses)

Data set designation	Genotype	Number of records (*N*)
**Pure breeds (randomised design)**		
	SAB	457
	ZB	74
	KR	50
**SAB and ZB ostriches and their reciprocal crosses (2 × 2 diallel crossbreeding design)**		
	SAB	519
	ZB	73
	ZB x SAB	105
	SAB x ZB	66
**SAB and KR ostriches and their reciprocal crosses (2 × 2 diallel crossbreeding design)**		
	SAB	371
	KR	52
	KR x SAB	114
	SAB x KR	67

Ostriches were slaughtered at a local European Union approved ostrich abattoir, according to standard South African techniques as described by Wotton and Sparrey (2002), Brand (2006) and Hoffman (2012). At the abattoir the ostriches were fasted for 24 h, electrically stunned, hoisted by the legs, and bled. Slaughter weights are routinely recorded at the abattoir after bleeding out and removal of most feathers. The skins were processed, graded and cured at the ostrich tannery of Cape Karoo International, Oudtshoorn, South Africa. Skin weight, skin thickness, crown traits, neckline traits and nodule traits were recorded afterwards on the processed skins (see Tables S1 and S2 for trait descriptions).

## Data analysis

Data was analysed using ASReml software (Gilmour et al. 2015). Linear models, including only fixed effects, were used in single-trait analyses to determine the significance using Wald F-statistics for each trait. Slaughter date was used as contemporary group. Sex, genotype, and the interaction between sex and genotype were modelled as fixed effects, while slaughter age was fitted as a linear covariate. The analyses were also repeated with live weight included as covariate to account for differences in live weight. Data from purebred SAB, ZB and KR birds represented in the same slaughter groups were analysed together. Data from SAB, ZB and their reciprocal crosses that were slaughtered together, but at different times between 2007 and 2020, were similarly analysed together and lastly, data from SAB, KR, and their reciprocal crosses that were slaughtered together were analysed to assess crossbreeding effects between the latter genotypes (see Table 1). Least significant differences protected by a significant F-value (*P* < *0.05*) in the ANOVA (Snedecor and Cochran 1968) were used to differentiate between genotypes.

The degrees of freedom for genetic group in the two sets of crossbreeding analyses were used to derive linear contrasts for the effects of additive breed, direct heterosis (the performance of the reciprocal cross relative to the mid-parent value of the pure breeds) and dam line or maternal additive effects (the performance of progeny from the two dam lines studied relative to each other).

## Results

The ostriches slaughtered ranged from 275 to 574 days of age. The mean and standard deviation for age at slaughter amounted to 378 ± 51 days in this study. The derived coefficients of variation (CV%) were widely variable, ranging from below 6% for crust skin size to more than 47% for hair follicle score (see Table S3 for descriptive statistics).

The significance of fixed effects for the pure breed analysis are shown in Table 2. The effect of slaughter group was significant (*P < 0.001*) for all the traits in the single-trait analyses of pure breeds (SAB, ZB and KR birds), as well as for the analyses of the SAB x ZB and the SAB x KR 2 × 2 diallel crossbreeding designs.

The effect of sex was significant for skin weight and crown length in the pure breed analysis, and for skin thickness in the crossbreeding analyses. Skins from males were significantly heavier than those of the females in all analyses (*P < 0.001*). A significant sex by genotype interaction was found only for skin weight (Table 2).

**Table 2:** Significance level of the fixed effects (slaughter group, sex and genotype) and interactions for the respective traits in the pure breed analysis

Traits	Slaughter group	Genotype		Sex	Sex x genotype interaction
Slaughter weight (kg)	***	***		n.s.	n.s.
Crust skin size (dm^2^)	***	***		n.s.	n.s.
Skin weight (kg)	***	***		***	*
Skin thickness (mm)	***	***		**	n.s.
Crown length (mm)	***	*		*	n.s.
Crown width (mm)	***	n.s.		n.s.	n.s.
Crown shape (mm)	***	***		n.s.	n.s.
Neckline total length (mm)	***	***		n.s.	n.s.
Neckline crown length (mm)	***	***		n.s.	n.s.
Neckline width top (mm)	***	n.s.		n.s.	n.s.
Neckline width middle (mm)	***	***		n.s.	n.s.
Nodule size score (1–9)	***	**		n.s.	n.s.
Nodule shape score (1–9)	***	n.s.		n.s.	n.s.
Hair follicle score (1–9)	***	*		**	n.s.

n.s.: not significant; * *P* < 0.05; ** *P* < 0.01; *** *P* < 0.001

### The comparison of pure breeds (SAB, ZB and KR)

Genotype significantly affected most of the skin traits measured (Table 3). ZB and KR birds outperformed their SAB contemporaries for most size-related traits (*P* < *0.05*). ZB birds had the highest (although not statistically different from KR birds) slaughter weight (93.8) and biggest skins (149.4 dm^2^). KR birds had the heaviest (1.098), and longest skins (902.1), although not statistically different from ZB birds. The means for ZB birds were higher than for SAB contemporaries for crown shape (*P < 0.05*); the mean for KR birds being intermediate and not different from either other strain. KR birds had higher scores than SAB contemporaries for nodule shape score (*P < 0.05*). No conclusive significant differences were found between ZB and KR birds, except for hair follicle score. KR birds were intermediate and not different from either other breed for crown shape (*P > 0.05*). The same situation applied to ZB birds for nodule shape where its mean was intermediate relative to the other breeds, but not significantly different from either (*P > 0.05*). For hair follicle score a significant breed effect was observed (*P < 0.05*), with the means for the ZB and SAB ostriches not differing significantly from each other, while the skins produced by KR birds had a significantly higher hair follicle score than the other genotypes (*P < 0.05*).

**Table 3:** Number of records (N) and predicted means (± standard errors) for ostrich slaughter and skin traits according to genotype for South African Black (SAB), Zimbabwean Blue (ZB) and Kenyan Red (KR) ostriches

Trait	SAB	ZB	KR	Significance
**Slaughter trait**	(*N* = 457)	(*N* = 73)	(*N* = 50)	
Slaughter weight (kg)	84.1 ± 0.7^a^	93.8 ± 1.3^b^	88.4 ± 1.6^b^	**
**Skin traits**	(*N* = 453)	(*N* = 73)	(*N* = 49)	
Crust skin size (dm^2^)	142.9 ± 0.5^a^	149.4 ± 0.9^b^	146.2 ± 1.1^b^	**
	(*N* = 376)	(*N* = 68)	(*N* = 47)	
Skin weight (kg)	0.928 ± 0.01^a^	1.054 ± 0.02^b^	1.098 ± 0.02^b^	**
	(*N* = 379)	(*N* = 69)	(*N* = 49)	
Skin thickness (mm)	0.90 ± 0.01^a^	0.95 ± 0.03^a, b^	1.01 ± 0.03^b^	*
**Crown and neckline traits**	(*N* = 379)	(*N* = 69)	(*N* = 49)	
Crown length (mm)	887.2 ± 3.2^a^	901.7 ± 6.1^b^	902.1 ± 7.1^b^	**
Crown width (mm)	668.7 ± 2.5	680.4 ± 4.7	672.5 ± 5.4	n.s.
Crown shape (mm)	304.9 ± 1.7^a^	319.77 ± 3.1^b^	310.9 ± 3.6^a.b^	*
Neck total length (mm)	616.5 ± 3.6^a^	659.1 ± 6.8^b^	656.9 ± 7.8^b^	**
Neck crown length (mm)	492.5 ± 2.8^a^	512.9 ± 5.2^b^	526.3 ± 6.0^b^	**
Neck width top (mm)	35.2 ± 0.4	36.2 ± 0.8	35.9 ± 0.9	n.s.
Neck width middle (mm)	17.7 ± 0.3^a^	20.5 ± 0.5^b^	20.2 ± 0.6^b^	**
**Subjective traits**	(*N* = 377)	(*N* = 69)	(*N* = 49)	
Nodule size score (1–9)	4.4 ± 0.1^a^	4.6 ± 0.1^b^	4.8 ± 0.1^b^	*
Nodule shape score (1–9)	4.1 ± 0.1^a^	4.2 ± 0.1^a.b^	4.5 ± 0.2^b^	*
Hair follicle score (1–9)	3.7 ± 0.1^a^	3.5 ± 0.2 ^a^	4.3 ± 0.2^b^	*

n.s.: not significant; * *P* < 0.05; ** *P* < 0.01

^a, b^ Denotes significant differences between means within a row (*P* < 0.05)

When farm weight (live weight measured on farm) was included in the analysis as a covariate (Table 4), the differences between the skin traits of SAB birds compared to ZB and KR contemporaries were reduced. The differences in slaughter weight of SAB ostriches compared to ZB and KR ostriches were also decreased, but it remained significant, however. The differences in skin size were reduced from 2.3% (KR) and 4.6% (ZB) to 1.1% for both when weight was included as a covariate (*P < 0.01*). The difference in skin weight from that of the SAB ostriches was reduced from 18 to 15% for KR ostriches and from 13 to 4.4% for ZB ostriches (*P < 0.01*). KR ostriches had the longest crown areas (nodulated area), and necklines within the crown area after live weight was factored in. The ZB and KR ostriches had wider necklines when measured in the middle of the crown area, however. KR ostriches had higher scores for nodule shape than ZB ostriches though, while the KR ostriches still had the highest hair follicle scores (*P* < 0.05).

**Table 4:** Number of records (N) and predicted means ± standard error for ostrich slaughter and skin traits according to genotype for South African Black (SAB), Zimbabwean Blue (ZB) and Kenyan Red (KR) ostriches (with farm weight included as a covariate)

Trait	SAB	ZB	KR	Significance
**Slaughter trait**	(*N* = 457)	(*N* = 73)	(*N* = 50)	
Slaughter weight (kg)	84.0 ± 0.3^a^	85.6 ± 0.6^b^	85.7 ± 0.7^b^	*
**Skin traits**	(*N* = 453)	(*N* = 73)	(*N* = 49)	
Crust skin size (dm^2^)	142.8 ± 0.3^a^	144.5 ± 0.6^b^	144.6 ± 0.7^b^	**
Skin weight (kg)	0.937 ± 0.01^a^	0.978 ± 0.01^b^	1.083 ± 0.02^b^	**
Skin thickness (mm)	0.90 ± 0.01	0.92 ± 0.03	1.00 ± 0.03	n.s.
**Crown and neckline traits**	(*N* = 376)	(*N* = 68)	(*N* = 47)	
Crown length (mm)	889.9 ± 2.9^a, b^	884.9 ± 5.7^a^	899.3 ± 6.3^b^	**
Crown width (mm)	670.7 ± 2.4	667.9 ± 4.4	670.3 ± 4.9	n.s.
Crown shape (mm)	306.2 ± 1.6^a^	312.6 ± 3.0^b^	309.8 ± 3.4^a.b^	*
Neckline total length (mm)	618.2 ± 3.6^a^	648.5 ± 6.8^b^	655.2 ± 7.5^b^	**
Neckline crown length (mm)	494.0 ± 2.6^a^	503.3 ± 5.2^a^	524.7 ± 5.7^b^	**
Neckline width top (mm)	35.4 ± 0.4	35.2 ± 0.8	35.8 ± 0.9	n.s.
Neckline width middle (mm)	17.80 ± 0.28^a^	20.04 ± 0.56^b^	20.11 ± 0.62^b^	**
**Nodule traits**	(*N* = 377)	(*N* = 69)	(*N* = 49)	
Nodule size score (1–9)	4.4 ± 0.1^a^	4.6 ± 0.1^a, b^	4.8 ± 0.1^b^	*
Nodule shape score (1–9)	4.2 ± 0.1^a.b^	4.1 ± 0.1^a^	4.5 ± 0.2^b^	*
Hair follicle score (1–9)	3.7 ± 0.1 ^a^	3.4 ± 0.2 ^a^	4.2 ± 0.2^b^	*

n.s.: not significant; * *P* < 0.05; ** *P* < 0.01

^a, b^ Denotes significant differences in means within a row (*P* < 0.05)

### SAB, ZB and their reciprocal crosses

For the SAB, ZB and their reciprocal crosses there were significant differences in slaughter weight, with the ZB x SAB cross being the heaviest at 92.7 kg (Table 5). A significant additive breed effect was observed for slaughter weight (10.4%), crust skin size (3.9%), skin weight (13.4%), crown length (1.5%), crown shape (4.7%), neckline total length (7.0%), neckline crown length (4.5%) and neckline width in the middle of the crown (16.4%).

Significant levels of heterosis were found for slaughter weight (4.3%), crust skin size (1.7%), skin weight (3.5%) and nodule size score (3.7%), respectively. Significant dam line effects were observed for skin weight (4.1%), crown shape (2.4%) and neckline width at the middle (4.5%). For hair follicle score, heterosis and the dam line effect amounted to 12% and 4.1% respectively. It should be noted that these effects are deemed negative (i.e. less desirable), however, due to hair follicles being undesirable because it visually detracts from leather quality.

**Table 5:** Number of records (N) and least squares means ± standard errors for ostrich slaughter and skin traits for South African Black (SAB), Zimbabwean Blue (ZB) ostriches and their reciprocal crosses. The significance of the additive breed effect, direct heterosis (H_d_) and dam line effects (H_m_) are also indicated for each trait

Trait	SAB	ZB	ZB x SAB	SAB x ZB	Breed	H_d_	H_m_
**Slaughter traits**	(*N* = 519)	(*N* = 72)	(*N* = 105)	(*N* = 66)			
Slaughter weight (kg)	82.5 ± 0.5	91.2 ± 1.3	92.7 ± 1.1	88.4 ± 1.3	**	***	n.s.
**Skin traits**	(*N* = 507)	(*N* = 72)	(*N* = 93)	(*N* = 65)			
Crust skin size (dm^2^)	141.1 ± 0.4	146.7 ± 0.9	147.7 ± 0.8	143.0 ± 0.9	**	***	n.s.
Skin weight (kg)	0.899 ± 0.01	1.019 ± 0.02	1.013 ± 0.02	0.972 ± 0.02	*	*	*
Skin thickness (mm)	0.938 ± 0.01	0.948 ± 0.02	0.992 ± 0.02	1.004 ± 0.02	n.s.	n.s.	n.s.
**Crown and neckline traits**	(*N* = 437)	(*N* = 67)	(*N* = 101)	(*N* = 65)			
Crown length (mm)	876.1 ± 2.5	889.6 ± 5.8	896.6 ± 4.7	871.8 ± 5.7	*	n.s.	n.s.
Crown width (mm)	664.7 ± 2.1	675.2 ± 4.8	681.8 ± 3.9	676.3 ± 4.8	n.s.	n.s.	n.s.
Crown shape (mm)	302.8 ± 1.3	317.1 ± 2.8	308.1 ± 2.3	308.4 ± 2.8	**	n.s.	*
Neckline total length (mm)	611.0 ± 2.7	653.8 ± 6.2	639.8 ± 5.0	621.4 ± 6.1	***	n.s.	n.s.
Neckline crown length (mm)	487.4 ± 2.2	509.1 ± 5.4	514.1 ± 4.4	501.2 ± 5.4	*	n.s.	n.s.
Neckline width top (mm)	34.5 ± 0.3	36.0 ± 0.8	37.7 ± 0.6	34.4 ± 0.8	*	n.s.	n.s
Neckline width middle (mm)	17.1 ± 0.2	19.9 ± 0.5	19.1 ± 0.4	17.9 ± 0.5	***	n.s.	*
**Subjective traits**	(*N* = 433)	(*N* = 67)	(*N* = 101)	(*N* = 65)			
Nodule size score (1–9)	4.3 ± 0.1	4.6 ± 0.1	4.8 ± 0.1	4.6 ± 0.1	*	*	n.s.
Nodule shape score (1–9)	4.3 ± 0.1	4.5 ± 0.1	4.5 ± 0.1	4.0 ± 0.1	*	n.s.	n.s.
Hair follicle score (1–9)	2.9 ± 0.1	2.9 ± 0.2	3.1 ± 0.2	3.4 ± 0.2	n.s.	*	*

n.s.: not significant; * *P* < 0.05; ** *P* < 0.01; *** *P* < 0.001

When live weight was included as a covariate all heterosis estimates became insignificant, with the exception of hair follicle score (*P* < 0.01), while only the dam line effect for crown shape remained significant at 1.7% but at a reduced level of significance *P < 0.05)*. For hair follicle score, the inclusion of weight as a covariate reduced heterosis to 11%, and the dam line effect to 3.8%. The SAB x ZB cross had a significantly higher mean score for hair follicles than either of the pure genotypes.

### SAB, KR and their reciprocal crosses

Significant genetic group effects were also observed for slaughter weight of the SAB, KR, and their reciprocal crosses (*P < 0.01*), with the mean for slaughter weight of the KR x SAB cross being the highest at 95.3 kg (Table 6). The significance of additive breed effects in Table 6 was largely consistent with those reported from the pure breed analyses (Table 3), with the exception of skin size and crown length. KR birds outperformed SAB’s for skin size and crown length in the pure breed analysis, but not in this analysis that included less SAB ostriches (Table 6). Only skin weight was significantly affected (*P* < 0.05) by breed combination, with SAB birds having lighter skins than their KR, KR x SAB and SAB x KR contemporaries. All the neckline traits were influenced by genetic group (*P < 0.05*), except for neckline width at the top. For nodule traits, genetic group effects were visible both for nodule size score (*P < 0.01*) and nodule shape score (*P < 0.05*), with KR x SAB birds having higher means for nodule size (4.75) and nodule shape (4.78).

Significant heterosis estimates were found for slaughter weight (5.7%), crust skin size (2.9%), crown length (1.7%), crown shape (2.7%) and nodule size score (4.4%). Progeny hatched from eggs produced by the KR as dam line had a better performance for slaughter weight (3.7%), neckline total length (3.9%), neckline crown length (3.4%), neckline width in the middle (5.6%) as well as nodule size score (3.8%). For hair follicle score, significant additive, heterosis and dam line effects were found, with SAB ostriches having a lower average score than their KR, KR x SAB and SAB x KR contemporaries.

**Table 6:** Number of records (N) and least squares means ± standard errors for ostrich slaughter and skin traits for South African Black (SAB), Kenyan Red (KR) ostriches and their reciprocal crosses. The significance of breed additive effect, direct heterosis (H_d_) and dam line effects (H_m_) are also indicated for each trait

Trait	SAB	KR	KR x SAB	SAB x KR	Breed	H_d_	H_m_
**Slaughter traits**	(*N* = 361)	(*N* = 50)	(*N* = 112)	(*N* = 65)			
Slaughter weight (kg)	86.8 ± 1.0	92.9 ± 1.6	95.3 ± 1.3	94.8 ± 1.5	**	**	*
**Skin traits**	(*N* = 367)	(*N* = 51)	(*N* = 110)	(*N* = 66)			
Crust skin size (dm^2^)	143.9 ± 0.5	147.5 ± 1.2	150.2 ± 1.0	149.5 ± 1.1	n.s.	*	n.s.
	(*N* = 311)	(*N* = 49)	(*N* = 111)	(*N* = 67)			
Skin weight (kg)	0.967 ± 0.01	1.15 ± 0.02	1.12 ± 0.02	1.07 ± 0.02	**	n.s.	n.s.
	(*N* = 315)	(*N* = 51)	(*N* = 113)	(*N* = 67)			
Skin thickness (mm)	0.88 ± 0.02	1.00 ± 0.03	0.94 ± 0.3	0.94 ± 0.03	n.s.	n.s.	n.s.
**Crown and neckline traits**	(*N* = 315)	(*N* = 51)	(*N* = 113)	(*N* = 67)			
Crown length (mm)	878.9 ± 4.4	897.1 ± 7.1	904.2 ± 5.7	902.6 ± 6.6	n.s.	*	n.s.
Crown width (mm)	668.2 ± 3.8	671.8 ± 6.1	676.8 ± 4.9	682.5 ± 5.6	n.s.	n.s.	n.s.
Crown shape (mm)	305.7 ± 2.2	314.3 ± 3.6	319.3 ± 2.9	317.7 ± 3.3	n.s.	*	n.s.
Neckline total length (mm)	628.3 ± 5.2	673.4 ± 8.4	654.8 ± 6.8	659.9 ± 7.8	**	n.s.	*
Neckline crown length (mm)	498.3 ± 4.3	534.9 ± 6.8	522.1 ± 5.5	520.2 ± 6.3	*	n.s.	*
Neckline width top (mm)	37.3 ± 0.6	38.1 ± 1.0	37.9 ± 0.8	37.8 ± 0.9	n.s.	n.s.	n.s.
Neckline width middle (mm)	18.2 ± 0.4	20.6 ± 0.6	19.9 ± 0.5	19.7 ± 0.6	**	n.s.	*
**Subjective traits**	(*N* = 313)	(*N* = 51)	(*N* = 67)	19.7 ± 0.6			
Nodule size score (1–9)	4.3 ± 0.1	4.7 ± 0.1	4.8 ± 0.1	4.7 ± 0.1	**	*	*
Nodule shape score (1–9)	4.3 ± 0.1	4.7 ± 0.2	4.8 ± 0.1	4.4 ± 0.2	*	n.s.	n.s.
Hair follicle score (1–9)	3.7 ± 0.2	4.7 ± 0.3	4.3 ± 0.2	4.4 ± 0.2	**	*	*

n.s.: not significant; * *P* < 0.05; ** *P* < 0.01

Inclusion of on-farm weight as a covariate reduced levels of heterosis for slaughter weight to 1.1%, for crust skin size to 0.7%, for crown length to 0.5% and for crown shape to 1.0%. Heterosis for nodule shape became insignificant (*P > 0.05*). The inclusion of weight as a covariate reduced the dam line effect for slaughter weight, neckline total length, neckline crown length, neckline width in the middle and nodule size to respectively 0.7%, 3.5%, 2.9%, 5.1% and 2.6%. For hair follicle score, the significant effects of additive breed, heterosis and dam line were reduced from 27%, 4.9% and 13–25%, 4.0% and 12%, respectively.

## Discussion

The average slaughter age for this study was higher than the average slaughter age of 322 ± 111 days reported by Engelbrecht (2013). This can be attributed to the inclusion of more birds of typical slaughter age (12 months and older) in this dataset. Slaughter group significantly (*P < 0.001*) affected all traits across all analyses, which are consistent with literature (Rauw and Gomez-Raya 2015; Engelbrecht 2013). Since such effects are a combination of climate, management, year and season, it is usually not repeatable. The data from the processed skins were collected from skins slaughtered over a period of 11 years, over which time one could expect that slaughter practices changed slightly. It is thus important to model such effects for the variation it explains, although these effects are often not easily explicable and without any real predictive value. It should also be considered when crossbreeding options are assessed that the ZB and KR genotypes were outperformed by the SAB for reproduction traits (Cloete et al. 2008, 2022).

### Pure breeds (SAB, ZB and KR)

It was clear that the ZB and KR ostriches outperformed SAB birds for slaughter weight and most size-related traits (Table 3). This confirms that both the ZB and KR wild genotypes are naturally larger and heavier than the locally developed SAB breed (Jarvis 1998; Cloete et al. 2008; Davids et al. 2010; Engelbrecht et al. 2008, 2018). The observed results pertaining to quantitative skin traits are attributed to the positive correlation between the size and/or weight of the animal and quantitative skin traits such as skin size that was reported previously by Engelbrecht (2013). Hair follicle score was the only trait that significantly differed between ZB and KR ostriches, with ZB ostriches having the lowest hair follicle scores (Table 3).

It is notable that no breed differences were found for skin thickness. Earlier studies on different genotypes of sheep (Oliveira et al. 2007; Teklebrhan et al. 2012; Ebrahiem 2015) and goats (Dal Monte et al. 2004; Oliveira et al. 2007) similarly reported an absence of significant breed effects for skin thickness. Unfortunately, no comparable results are available for other ratite species.

ZB and KR ostriches had longer crowns compared to SAB ostriches. This result arguably stems from the higher live weight and larger skin size of ZB and KR birds. Significant additive breed effects were seen for neckline total length, neckline crown length and neckline width in the middle of the crown, with ZB and KR ostriches also having longer and wider necklines than the SAB ostriches. However, there were a marked reduction and even elimination of some breed effects when live weight was added as a linear covariate to the models.

Scores for nodule size were higher for the ZB (4.6) and KR (4.8) strains compared to the SAB ostriches (4.4). This result could be attributed to the higher weight of the former strains, given the positive genetic and phenotypic correlations of live weight with nodule size (Engelbrecht 2013).

According to these results, it therefore seems that leather from ZB and KR ostriches would be acceptable and perhaps even more valuable than those from the SAB ostriches, because the appearance (size and shape) of the nodules directly influences the quality and price of the skin. This is because the nodulated part (the crown area) is the valuable part of the ostrich skin, as the nodules are key to differentiating ostrich leather from other comparable products. However, the lower hair follicle scores (more acceptable) for the ZB (3.5) and SAB (3.7) strains are preferable to the higher mean score reported for KR ostriches (4.2; Table 3).

### SAB, ZBs and their reciprocal crosses

Additive breed effects in this analysis were mostly like those presented in Table 3 (see Table 5). These effects will thus not be discussed again. The reciprocal cross of the SAB and ZB strains outperformed the pure breeds, resulting in significant heterosis estimates for live weight and other size-related traits. These results suggest that it would be viable to combine the relatively high live weight of ZB with the better reproduction of the SAB for economic gain in a commercial crossbreeding system as suggested by Cloete et al. (2008). Chick survival, which is often considered as a constraint to commercial ostrich production, also benefitted in crossbred chicks produced in this way (Essa and Cloete 2006; Engelbrecht et al. 2008), with ZB x SAB (0.23 ± 0.02) crosses showing lower mortality rates than ZB (0.38 ± 0.03), SAB x ZB (0.34 ± 0.03) and the SAB chicks (0.27 ± 0.01) (Engelbrecht et al. 2008). Hoffman et al. (2007) accordingly reported that crossing ZB and SAB ostriches improved live weight and carcass yield of the hybrid, although this study was based on a very limited number of birds. The derived heterosis estimates demonstrated that improved body weight in hybrids relative to the mid parent value can be achieved via crossbreeding (Davids et al. 2010; Engelbrecht et al. 2008, 2018), which also resulted in larger skins in this study.

The introduction of the heavier genotypes influenced crust skin size and skin weight, identifying the exploitation of heterosis by crossbreeding as a viable means to improve ostrich skin yield in a commercial crossbreeding system as has commonly been reported for other species. A crossbreeding study by Theunissen et al. (2017) on beef cattle also showed that the introduction of exotic breeds resulted in improvement of leather yield and quality.

The breed and crossbreeding effects for crown and neckline traits of SAB, ZB and their reciprocal crosses could not be substantiated from the literature, since no known previous crossbreeding studies investigated these traits that are unique to ostrich leather. No heterosis was found for nodule shape score although the absolute mean scores of the crossbred (4.5 for ZB x SAB and 4.0 for SAB x ZB) birds seemed higher (indicating bigger nodules). This result should be investigated further with more animals to allow for the estimation of means with smaller standard errors.

The lack of heterosis in some traits showed that crossing the ZB with SAB resulted in crossbred combinations with values on or close to the mid-parent value for such traits. Although dam line effects were absent for the nodule traits (Table 5), the presence of heterosis for nodule size showed that it is feasible to improve this trait in crossbred progeny.

The only trait where estimates of non-additive genetic variation was significant (*P < 0.05*), but in an unfavourable direction, was hair follicle score. Crosses between the SAB and ZB, as well as with a ZB dam were inferior to the pure breeds and progeny with a SAB dam. Crossbreeding of these genotypes is therefore not recommended for addressing the occurrence of hair follicles. This result could not be substantiated from the literature as no other studies on skin traits of these breed combinations could be sourced, however. Further investigation is indicated.

### SAB, KRs and their reciprocal crosses

The crossbreeding of SAB with KR ostriches resulted in heterosis and dam line effects for slaughter weight, with crossbred animals outperforming purebreds and progeny with a KR dam outperforming the progeny of SAB dams (Table 6). These results shows that the body weight of ostriches can be improved above the mid-parent values by crossbreeding the domesticated SAB strain with KR ostriches. Similar estimates of heterosis for live weight have been reported for goats (Afolabi et al. 2017), sheep (Iwan 1971) and cattle (Theunissen 2011). The only skin trait that was subject to a significant level of non-additive genetic variation was skin size, where the crossbred progeny outperformed the mid-parent value of the pure breeds (Table 6). This advantage in skin size was also transferred to crown length and crown shape, but not to the neckline traits. Another size-related trait, namely nodule size score, also exhibited significant heterosis (*P* < 0.05). As for the crosses between the SAB and ZB birds, hair follicle score was subject to unfavourable heterosis as a high occurrence of hair follicles was observed for the crosses compared to the SAB strain, which negatively affects skin quality.

Progeny with a KR dam had a higher live weight than their contemporaries with a SAB dams. This advantage was not transferred to skin size, but neckline was unfavourably affected (i.e. were larger), which negatively impacts the cutting value of the skin (*P < 0.05*) in progeny of KR dams compared to offspring produced by SAB dams. In contrast, nodule size scores of progeny descended from KR dams were improved (*P < 0.05*) compared to descendants of SAB dams. This result can be attributed to the larger size of KR (live weight of 92.9 vs. 86.8 kg of SAB birds) and the known positive correlation between nodule size scores and size (Engelbrecht et al. 2005, 2009; Engelbrecht 2013). Nodule size may thus be improved by crossbreeding with larger breeds. As for the crosses between the SAB and the ZB birds, hair follicle score was unfavourably affected (higher) in the progeny of KR dams (*P* < 0.05).

## Conclusions

This study revealed that the Zimbabwean Blue (ZB) and Kenyan Red (KR) birds performed better for weight and size-related skin traits than their South African Black (SAB) contemporaries. Crossbreeding could therefore be expected to improve slaughter weight and other size-related traits of hybrids over the mid-parent value of purebreds. Apart from higher hair follicle score for KR birds, subjective skin quality traits for ZB, KR birds and their crosses, were better or comparable to values recorded for the SAB breed. The study showed that implementation of crossbreeding can deliver economic advantages to commercial farmers and the entire ostrich industry with regards to most skin traits. Caution should, however, be exercised when considering the introduction of KR ostriches into commercial ostrich flocks, due to the higher hair follicle scores reported here for KR birds and their crossbred progeny. The smaller number of birds and available records for these birds could possibly have played a role in this result.

Furthermore, decisions on breed substitution and crossbreeding should hinge on the effects on reproduction and feather production. This study should therefore be followed by comprehensive studies on traits complexes that includes all other traits of economic importance. This will inform the ostrich industry on how to best make use of all avenues and options of pure breeding as well as crossbreeding to ensure optimal commercial success. Based on the current prizes for day-old chicks and ostrich feathers, however, it is foreseen that farming with the SAB breed will stay the number one choice for local ostrich producers, whom also must consider the demeanour of the birds with regards to ease of handling and proneness to injuries of the wild genotypes.

## Electronic supplementary material

Below is the link to the electronic supplementary material.

Supplementary Material 1

## Data Availability

The datasets generated and/or analysed during the current study are not publicly available due to source privacy and confidentiality but are available from the corresponding author on reasonable request.
