# Supplementary material for: Estimation of breed effects and non-additive genetic variation for ostrich slaughter and skin traits
Source: Trop Anim Health Prod. 2024 Sep 30;56(8):306. doi: 10.1007/s11250-024-04168-8 (PMC11442555; doi:10.1007/s11250-024-04168-8)
Supplement: Supplementary file 1 — Supplementary Material 1 [file 11250_2024_4168_MOESM1_ESM.docx]

**Estimation of breed effects and non-additive genetic variation for ostrich slaughter and skin traits**

**Tropical Animal Health and Production**

**Khetho Ratshilumela Nemutandani^1,2#^, Anel Engelbrecht^3^, Schalk Willem Petrus Cloete^1^, Kennedy Dzama^1^ & Obert Tada^2^**

^1^Department of Animal Sciences, Stellenbosch University, Private Bag X1, Matieland 7602, South Africa, ^2^Department of Agricultural Economics and Animal Production, University of Limpopo, Private Bag X1106, Sovenga 0727, South Africa,

^3^Directorate Animal Sciences, Western Cape Department of Agriculture, Private Bag X1, Elsenburg 7607, South Africa. [khetho.nemutandani@ul.ac.za](mailto:khetho.nemutandani@ul.ac.za)

**Table S1** Quantitative traits that were objectively measured to evaluate the effect of breed and crossbreeding on slaughter and leather traits

| **Trait** | **Description** | **Unit of measurement** |
| --- | --- | --- |
| Farm weight | The live weight measured on farm, before pre-slaughter isolation (approximately 14 days before slaughter) | kg |
| Slaughter weight | The weight of the bird after stunning, exsanguination, and removal of feathers | kg |
| Crust skin size | The size of the processed skin measured at the tannery | dm^2^ |
| Skin weight | The weight of the skin after curing, processing, and preservation | kg |
| Skin thickness | Average of three skin thickness measurements taken on different places on the right flank using a leather thickness gauge | mm |
| Crown length | The length of the nodulated area, measured from the thinnest nodulated area at the base of the neck to the end of the rump | mm |
| Crown width | Distance between the two most distant nodules across the middle of the skin at the broadest area of the crown | mm |
| Crown shape | Distance (radius) between the center of the skin at the broadest area of the crown and the uttermost nodule on the outside edge of the crown at an approximate 30° angle | mm |
| Neckline total length | Total length of the neckline area on the back of the ostrich | mm |
| Neckline crown length | Neckline length within the crown area | mm |
| Neckline width top | Neckline width at the top of the crown | mm |
| Neckline width middle | Neckline width in the middle of the crown | mm |

kg, kilogram; mm, millimetres

**Table S2** Description of subjectively scored qualitative skin traits that were scored on a linear defined scale

| **Trait** | **Description** | **Score range** |
| --- | --- | --- |
| Crust skin grade | The grade allocated to the skin at the tannery based on the presence and quantity of visible defects and lesions, with 1 awarded to skins with the least damage and 5 to skins with the most damage | 1-5 |
| Nodule size score | Refers to the size of the feather nodules on the processed skin, with very small nodules (poor) scored as 1 and very big nodules scored as 9 | 1-9 |
| Nodule shape score  Hair follicle score | Refers to the shape of the feather nodules on the processed skin, with 1 indicating mostly poorly developed, open, or elongated nodules and 9 indicating well-rounded nodules  The overall occurrence of hair follicles in the crown area were scored, with one indicating no hair follicles and 9 indicating excessive occurrence of clearly visible hair follicles. | 1-9  1-9 |

**Table S3** Descriptive statistics for the available ostrich slaughter and skin traits, encompassing number of records (N), means, standard deviations (SD), coefficients of variation (CV%), kurtosis, skewness and range, utilizing all available data across genotypes, as collected from 2009 to 2020.

| **Trait (Unit of measurement)** |  | **N** | **Mean ± SD** | **CV%** | **Kurtosis** | **Skewness** | **Range** |
| --- | --- | --- | --- | --- | --- | --- | --- |
| Farm weight (kg) |  | 1137 | 101.4 ± 13.9 | 14 | 0.45 | -0.12 | 44.5-141.0 |
| Slaughter weight (kg) |  | 1116 | 87.1 ± 12.0 | 14 | 0.41 | -0.05 | 43.4-123.0 |
| Crust skin size (dm^2^) |  | 1104 | 143 ± 9 | 6 | 2.90 | -0.47 | 87-170 |
| Skin weight (kg) |  | 1016 | 0.974 ± 0.195 | 20 | 0.44 | 0.34 | 0.343-1.721 |
| Skin thickness (mm) |  | 1033 | 0.97 ± 0.33 | 34 | 1.19 | 1.04 | 0.29-2.17 |
| Crust skin grade (1-5) |  | 1102 | 2.9 ± 1.0 | 34 | -0.82 | -0.28 | 1-5 |
| Crown length (mm) |  | 1033 | 888 ± 71 | 8 | 1.37 | 0.58 | 558-1164 |
| Crown width (mm) |  | 1033 | 672 ± 39 | 6 | 6.96 | 1.27 | 546-966 |
| Crown shape (mm) |  | 1032 | 307 ± 24 | 8 | 2.29 | 0.02 | 194-399 |
| Neckline total length (mm) |  | 1033 | 628 ± 55 | 9 | 2.95 | 0.21 | 374-948 |
| Neckline crown length (mm) |  | 1033 | 503 ± 47 | 9 | 2.57 | -0.15 | 214-755 |
| Neckline width top (mm) |  | 1033 | 36 ± 6 | 18 | -0.11 | 0.19 | 18-56 |
| Neckline width middle (mm) |  | 1032 | 18 ± 5 | 26 | 1.26 | 0.77 | 7-42 |
| Nodule size score (1-9) |  | 1026 | 4.6 ± 1.0 | 22 | 0.26 | 0.32 | 2-8 |
| Nodule shape score (1-9) |  | 1026 | 4.3 ± 1.2 | 27 | 0.08 | 0.40 | 1-9 |
| Hair follicle score (1-9) |  | 1025 | 3.6 ± 1.7 | 47 | -0.02 | 0.57 | 1-9 |
